# E-Beam Patterned Gold Nanodot Arrays on Optical Fiber Tips for Localized Surface Plasmon Resonance Biochemical Sensing

**DOI:** 10.3390/s101009397

**Published:** 2010-10-20

**Authors:** Yongbin Lin, Yang Zou, Yuanyao Mo, Junpeng Guo, Robert G. Lindquist

**Affiliations:** 1 Nano and Micro Device Center, University of Alabama in Huntsville, Huntsville, AL 35899, USA; E-Mails: yang.zou@uah.edu (Y.Z.); moy@uah.edu (Y.M.); jguo@eng.uah.edu (J.G.); 2 Department of Electrical and Computer Engineering, University of Alabama in Huntsville, Huntsville, AL 35899, USA; E-Mail: lindquis@eng.uah.edu

**Keywords:** localized surface plasmon resonance sensor, nanofabrication, fiber optic biosensor

## Abstract

Electron beam lithography (EBL) was used to directly pattern periodic gold nanodot arrays on optical fiber tips. Localized surface plasmon resonance of the E-beam patterned gold nanodot arrays on optical fiber tips was utilized for biochemical sensing. The advantage of the optical fiber based localized surface plasmon resonance (LSPR) sensors is the convenience to work with and work in harsh environments. An optical fiber tip LSPR refractive index sensor of 196 nm per refractive index unit (RIU) sensitivity has been demonstrated. The affinity sensing property of the fiber tip sensor was demonstrated using biotin/streptavidin as the receptor/analyte. The detection limit for streptavidin was determined to be 6 pM.

## 1. Introduction

Noble metal nanodots have been the subject of many research activities, due to their unique optical property of supporting localized surface plasmon resonances (LSPR). In the plasmon resonance condition, the metal nanodots exhibit strong electromagnetic field enhancement and energy confinement in the near field, therefore it is highly sensitive to the local refractive index change. On planar substrates, both metal nanoparticles [1–7] and nanoholes in thin metal films [8–12] have been investigated for high sensitivity chemical and biological sensors in the past.

The development of optical fiber based localized surface plasmon sensors for compact and label-free biochemical detections has been reported using various types of metal nanostructures [3–18]. Among them, chemically synthesized metal nanoparticles were immobilized on optical fiber tips as active sensing materials providing LSPR signals. For example, Mitsui *et al.* have demonstrated the red-shift of the LSPR wavelength of gold nanoparticles immobilized on the tip of a multimode optical fiber [14]. However, the reproducible immobilization of nanoparticles of identical size and arrangement is challenging, and stabilization is required against aggregation of metal nano-particles during the processes prior to immobilization on optical fiber tips. Fabrication of well arranged nanostructure arrays on fiber tips was reported by Smythe *et al.* [18]. The array of nano-scale optical antennas was first fabricated on a planar silicon substrate via EBL, and subsequently transferred to a silica fiber end facet using a thin sacrificial thiol-ene film that can be stripped from the Si substrate. A major challenge for either immobilization technique or transfer method reported earlier is that the adhesive strength of nano-particles to the fiber end facet is too low to endure even weak sonication, tape stripping, or a mild version of piranha cleaning. It was reported earlier that focused-ion-beam (FIB) milling can be used to define gold nanostructures on the tip of optical fiber [8,9]. However, FIB milling of gold film results in unwanted doping of the silica and gold with gallium ions, which changes the optical properties of the metal [19].

In this paper, we first describe an e-beam lithography nano-fabrication process that can directly pattern gold nanodot arrays on flatly cleaved optical fiber facets. Then we report an optical refractive index sensor based on the localized surface plasmon resonance of the gold nanodot array on the fiber tip. Affinity-based biological and chemical sensing was also demonstrated using the biotin/streptavidin system as model receptor and analyte, respectively. Our optical fiber LSPR sensor could be cleaned in piranha solution and reused more than five times in our experiments. The significance of our e-beam directly patterned fiber tip LSPR biochemical sensors is that they are robust and reusable because the Au layer was directly sputtered onto the optical fiber facet with strong adhesion.

## 2. Experimental Methods

### 2.1. Device Fabrication

**Figure 1.**
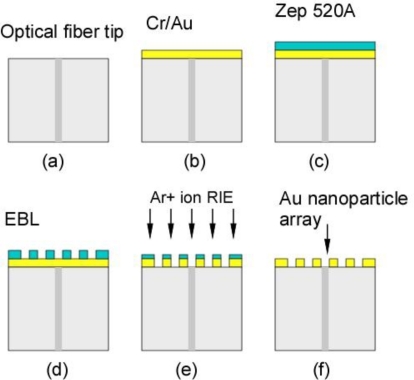
Illustration of fabrication process for gold nanodots array on the optical fiber tip.

We used standard optical communication fibers (Corning SMF-28) to make fiber surface plasmon sensors. The diameter of the fiber core is 9 μm, and the diameter of the fiber cladding is 125 μm. Figure 1 illustrates the fabrication process for Au nanoparticle array on an optical fiber tip [20]. The outer buffer coating on the optical fiber was removed using a stripper, and the fiber tip was cleaved perpendicularly to get a flat end face. A chromium (Cr) layer of 2 nm thickness was first deposited on the cleaved flat fiber tip as an adhesion layer by vacuum sputtering method. Then a 55 nm gold film is deposited on top of Cr layer in the same chamber. Electron beam (e-beam) resist (ZEP 520A, Zeon Chemicals) was coated on the fiber tip by a “dip and vibration” coating technique described below. To get an optimized ZEP thickness, we diluted the ZEP 520A with anisole solvent at the ratio 1:1. Immediately after dipping in the diluted ZEP solution, the fiber tip was gently taken out, held by a fiber clamp and put in the straight upward position. We use a method called “vibration coating” to get rid of extra liquid e-beam resist and achieve a uniform thickness coating layer on the facet of optical fiber end face. The vibration frequency and strength is controlled by the length of fiber tip outside of the fiber clamp and the initial displacement of the fiber tip. After the dip and vibration coating, the fiber tip was baked in an oven at 120 °C for 30 minutes. The resulting ZEP layer thickness was found to be around 80 nm to 100 nm. The EBL process was used to create the two dimensional nanoparticle array pattern in the e-beam resist. The voltage used was 30 kV and the dose was 70 μC/cm^2^. After e-beam exposure, the fiber tip was developed by dipping in the e-beam resist developer (ZEP N50) for 1 minute, followed by DI water rinse for 1 minute. The sample was dried by a pure nitrogen stream and baked in the oven at 120 °C for 30 minutes for dehydration and hardening.

The nanodots array pattern in the e-beam resist was transferred to Au layer by RIE etch with argon ions. In order to make ZEP film strong enough for the RIE etch process, we flood expose the ZEP film with an electron beam at 5 kV. The etch time was 3.5 minutes with 200 W platen power and argon (Ar) gas flow rate of 20 sccm. The remaining ZEP film was stripped by dipping overnight in the ZEP remover.

**Figure 2.**
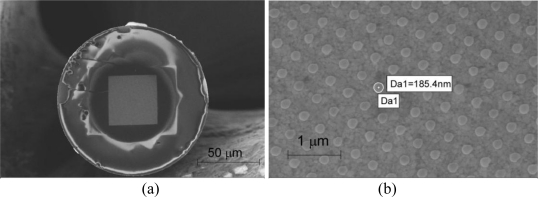
Scanning electron micrographs of a gold nanodot array on an optical fiber tip. **(a)** Overview of the optical fiber end facet, and **(b)** gold nanodot array on the optical fiber facet.

The SEM image of the Au nanoparticle array on optical fiber tip is shown in Figure 2(a). The fabricated area of Au nanoparticle array is 40 μm by 40 μm square. The periodicity of the array is 400 nm; the size of the Au nanodots is 185 nm in diameter, and 55 nm in height. The circle in the fiber tip is attributed to the e-beam resist film thickness variation due to edge bead. The ZEP film thickness inside of the circle is uniform. The diameter of the uniform thickness area is found to be around 60–80 μm, which is large enough to cover the fiber mode diameter (∼10 μm). Figure 2(b) shows the zoomed-in image of the Au nanoparticle array on the optical fiber tip.

### 2.2. Measurement Setup

**Figure 3.**
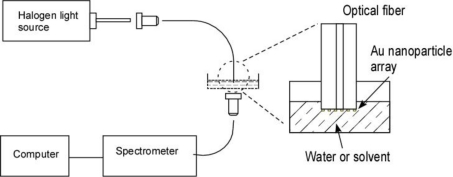
Optical setup for the fiber tip localized surface plasmon resonance sensor measurement.

Figure 3 illustrates the optical setup for the optical spectrum characterization. The light source used is a halogen lamp illuminator with a fiber optic bundle output. The light is coupled into freshly cleaved end of the single mode sensor fiber using a 10X microscope objective lens. Optical transmission through the array of nanoparticles on the tip of the sensor fiber is measured by a USB-2000 spectrometer (Ocean Optics Inc., USA). A fiber optical collimator and a multi-mode fiber are used to collect and transport the transmitted light to the spectrometer.

Using the fiber tip LSPR sensor, we were able to monitor the binding of streptavidin to the gold nanodots surfaces functionalized with a monolayer of biotin, which was deposited onto the gold nanodots surface via a covalent bond between biotinylated thiol group and the Au [21,22]. These results represent new applications in biochemical research and medical diagnostics.

### 2.3. Materials

Single mode silica optical fiber (SMF-28) was obtained from Corning Incorporated, USA. Acetone, isopropyl alcohol (IPA), methanol, and ethanol were purchased from Fisher Scientific, USA. All of the chemicals used here were of analytical reagent grade. Biotinylated polyethylene glycol (PEG) alkane thiol was purchased from Nanoscience Instruments, USA. Affinity purified streptavidin was purchased from Thermo Scientific, USA. All the biological reagents were kept at 4 °C. All aqueous solutions were prepared with purified water with a specific resistance of 18 MΩ cm. Piranha solution (30% H_2_O_2_/70% H_2_SO_4_) was used in chemical cleaning process. Piranha solution is a highly hazardous and reactive solution that may explode on coming into contact with organic solvents. Extreme precaution should be taken at all times.

## 3. Results and Discussion

### 3.1. Bulk Refractive Index Change Characterizations

**Figure 4.**
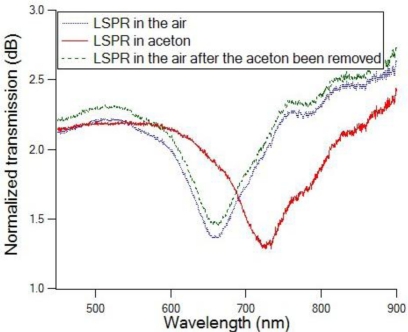
The transmission spectra of the fabricated fiber sensor in the air (blue dot and green dash) and in acetone (red solid).

The transmission spectrum of the Au nanodot array on the tip of single mode optical fiber is shown in Figure 4. The blue dotted curve and the green dash curve represent the fiber sensor LSPR spectra in air before and after being immersed in acetone. We intentionally adjusted the alignment to separate the blue dotted curve and the green dash curve to make them more visible. The red solid curve represents the fiber sensor LSPR in the acetone environment. An intense LSPR extinction dip is observed at 656 nm for the fiber sensor in air, and red shift for about 70 nm when the sensor tip dielectric environment changed from air to acetone. It can also be observed from Figure 4 that when the sensor fiber tip was removed from acetone and dried, the transmission spectrum went back to the original spectrum taken in air. The repeatability of this experiment is excellent (more than 10 times) for acetone and other solvents such as IPA and methanol. This fiber tip probe responds to the refractive index change in real time. The sensor can be reused if no biochemical residue is left on the tips after each measurement.

**Figure 5.**
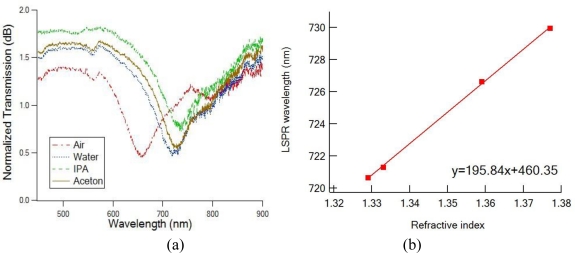
**(a)** Measured transmission spectra for the fiber sensor in various solvents. **(b)** Dependence of the LSPR peak wavelength on the index of refraction of the solvents.

The effect of varying refractive index of the dielectric environment surrounding the fiber tip sensor region was investigated by taking transmission measurements of the fiber sensors in air (n = 1), methanol (n = 1.329), water (n = 1.333), acetone (n = 1.359) and IPA (n = 1.377). These transmission spectra are shown in Figure 5. The measured LSPR wavelength λ_min_ moved toward longer wavelengths as the refractive index of the solvent increased. When the LSPR λ_min_ for each solvent was plotted *versus* the refractive index, a straight line could be fitted to the experimental points. A linear relationship between the shift of maximum extinction in the optical transmission spectrum and the change of refractive index of the surrounding medium was obtained, with the sensitivity of 196 nm/RIU. This sensitivity is comparable to reported sensitivity for Au nanoparticle array on planar substrate (∼200 nm/RIU) [5].

### 3.2. Biotin/Streptavidin for Affinity Sensing Characterizations

To demonstrate the effectiveness of the fiber tip LSPR sensor in affinity-based biological and chemical sensing, the biotin/streptavidin system was chosen as model receptor and analyte, respectively. The fiber tips are prepared for biosensing in the following way. First, the surface of the Au nanodots is cleaned two times by soaking in piranha solution for one minute and then rinsed with DI water for 2 minutes. Then the optical fiber tip LSPR sensors are functionalized with biotinylated PEG alkane thiol (concentration of 1 mM in anhydrous ethanol) for 24 hours in the room temperature. A monolayer of biotin is deposited onto the gold nanodots surface via a covalent bond between biotinylated thiol group and the Au. After that, the sample is rinsed by DI water and dried by nitrogen to remove the unbound molecules. Functionalized Au nanodots on the optical fiber tip are now ready for streptavidin detection, and are incubated in 200 μL of straptavidin in water at room temperature for 10 minutes. Then the fiber tip LSPR sensor is rinsed with DI water and dried using nitrogen. After each step, the transmission spectrum is measured in the air after the fiber tip is completely dried. We varied the concentration of streptavidin between 10^−13^ M and 10^−6^ M and measured the shift of LSPR wavelength after adsorption of streptavidin, respectively.

**Figure 6.**
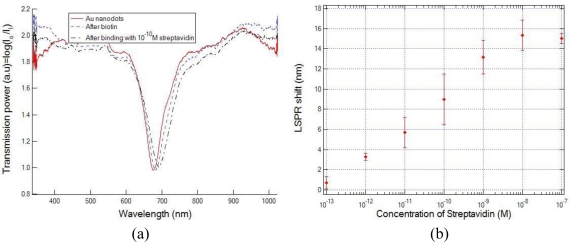
**(a)** Transmission spectrum and **(b)** plasmon peak shift due to the specific binding of streptavidin to the biotin on the Au nanodot surface on the optical fiber end facet. The error bars represent the standard deviation calculated from three independent measurements.

Figure 6(a) shows the transmission spectra measured during the biotin/streptavidin binding experiments. The concentration response of the sensor based on biotin/streptavidin binding on the Au nanodots surface is shown in Figure 6(b). The limit of detection (LOD), described as the lowest concentration for clear identification of wavelength shift, considering the errors and instrument noise, is determined to be 6 pM. In this case, the corresponding wavelength shift is about 5.4 nm, which is close to twice of the maximum standard deviations [error bars in Figure 6(b)], and can be easily identified considering all possible measurement errors and noise. For streptavidin concentrations higher than 10^−8^ M, the binding of biotin and streptavidin is saturated, subsequently no further wavelength shift is observed.

### 3.3. Numerical Simulation

**Figure 7.**
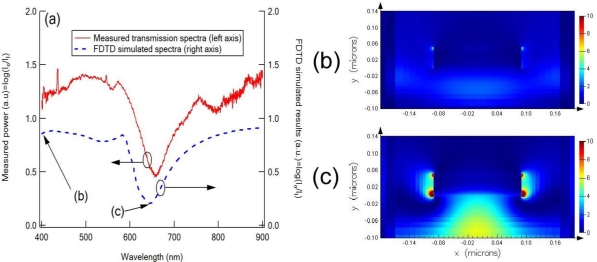
The measured and simulated transmission spectra for the nanodot array in air **(a)**, and the intensity of the electric field around the nanodots for 400 nm **(b)** and 650 nm **(c)**.

To physically illustrate the optical properties of the e-beam fabricated nanodot array on the fiber tip, we conducted the electromagnetic simulations using commercial finite-difference time-domain (FDTD) software (Lumerical Solution Inc., Canada). We investigated the electric field enhancement near the Au nanodots on the optical fiber tip for various wavelengths. The geometry of the Au nanodots array used in the modeling is a square array with 400 nm periodicity and each nanodot has disk-like shape with 55 nm thickness and 185 nm diameter. Drude-Lorentz model was used to describe the optical properties of gold metal. The calculated loss spectra for nanodots array in air have a LSPR wavelength at 650 nm (Figure 7a). The intensity of the electric field around the nanodots is shown in Figures 7(b,c) for the 400 nm and 650 nm wavelengths, respectively. The electric field intensity at 650 nm is much stronger than the electromagnetic field intensity at 400 nm because of the localized surface plasmon resonance in periodic array of nanodots at 650 nm wavelength.

## 4. Conclusions

In summary, we report an e-beam lithography nano-fabrication process to directly fabricate gold nanodot arrays on the facets of standard optical communication fibers. By utilizing the localized surface plasmon resonance of the gold nanodot arrays patterned on optical fiber tips, we demonstrated a refractive index sensor with the sensitivity of 196 nm/RIU. Optical fiber tip biochemical sensors were also demonstrated using the biotin/streptavidin system, and the limit of detection was determined to be 6 pM of streptavidin. Our result shows that gold nanodot arrays on optical fiber tips can be used as a high-sensitivity probe for chemical and biological analytes.

## References

[1] Mock J, Smith DR, Schultz S (2003). Local refractive index dependence of plasmon resonance spectra from individual nanoparticles. Nano Lett.

[2] Eah S, Jaeger H, Scherer N, Wiederrecht G, Lin X (2005). Plasmon scattering from a single gold nanoparticle collected through an optical fiber. Appl. Phys. Lett.

[3] Fu J, Park B, Zhao Y (2009). Limitation of a localized surface plasmon resonance sensor for Salmonella detection. Sens. Actuat. B: Chem.

[4] Yamamichi J, Iida M, Ojima T, Handa Y, Yamada T, Kuroda R, Imamura T, Yano T (2009). The mesoscopic effect on label-free biosensors based on localized surface plasmon resonance of immobilized colloidal gold. Sens. Actuat. B: Chem.

[5] Haynes C, Van Duyne R (2001). Nanosphere lithography: A versatile nanofabrication tool for studies of size-dependent nanoparticle optics. J. Phys. Chem. B.

[6] Sung J, Hicks E, Van Duyne R, Spears K (2007). Nanoparticle spectroscopy: Dipole coupling in two-dimensional arrays of L-shaped silver nanoparticles. J. Phys. Chem. C.

[7] Storhoff J, Elghanian R, Mucic R, Mirkin C, Letsinger R (1998). One-pot colorimetric differentiation of polynucleotides with single base imperfections using gold nanoparticle probes. J. Am. Chem. Soc.

[8] Dhawan A, Gerhold M, Muth J (2008). Plasmonic structures based on subwavelength apertures for chemical and biological sensing applications. Sens. J. IEEE.

[9] Dhawan A, Muth J, Leonard D, Gerhold M, Gleeson J, Vo-Dinh T, Russell P (2008). Focused ion beam fabrication of metallic nanostructures on end faces of optical fibers for chemical sensing applications. J Vac Sci Technol B.

[10] Prikulis J, Hanarp P, Olofsson L, Sutherland D, Käll M (2004). Optical spectroscopy of nanometric holes in thin gold films. Nano Lett.

[11] Gao D, Chen W, Mulchandani A, Schultz J (2007). Detection of tumor markers based on extinction spectra of visible light passing through gold nanoholes. Appl. Phys. Lett.

[12] Dahlin A, Zäch M, Rindzevicius T, Käll M, Sutherland D, Höök F (2005). Localized surface plasmon resonance sensing of lipid-membrane-mediated biorecognition events. J. Amer. Chem. Soci.

[13] Chang Y, Chen Y, Kuo H, Wei P (2006). Nanofiber optic sensor based on the excitation of surface plasmon wave near fiber tip. J. Biomed. Opt.

[14] Mitsui K, Handa Y, Kajikawa K (2004). Optical fiber affinity biosensor based on localized surface plasmon resonance. Appl. Phys. Lett.

[15] Gu C, Zhang Y, Schwartzberg A, Zhang J (2005). Ultra-sensitive compact fiber sensor based on nanoparticle surface enhanced Raman scattering. Proc. SPIE.

[16] White D, Stoddart P (2005). Nanostructured optical fiber with surface-enhanced Raman scattering functionality. Opt. Lett.

[17] Viheriala J, Niemi T, Kontio J, Rytkonen T, Pessa M (2007). Fabrication of surface reliefs on facets of singlemode optical fibres using nanoimprint lithography. Electron. Lett.

[18] Smythe E, Dickey M, Bao J, Whitesides G, Capasso F (2009). Optical antenna arrays on a fiber facet for *in situ* surface-enhanced Raman scattering detection. Nano Lett.

[19] Fu Y, Bryan N (2005). Investigation of physical properties of quartz after focused ion beam bombardment. Appl. Phys. B.

[20] Lin Y, Guo J, Lindquist R (2009). Demonstration of an ultra-wideband optical fiber inline polarizer with metal nano-grid on the fiber tip. Opt. Express.

[21] Barbillon G, Bijeon J, Plain J, de la Chapelle M, Adam P, Royer P (2007). Biological and chemical gold nanosensors based on localized surface plasmon resonance. Gold Bull.

[22] Yeri A, Gao L, Gao D (2010). Mutation screening based on the mechanical properties of DNA molecules tethered to a solid surface. J. Phys. Chem. B.

